# Low-cost drum granulator for mechanized seedball production

**DOI:** 10.1016/j.ohx.2023.e00397

**Published:** 2023-01-23

**Authors:** Sebastian Romuli, Achim Jesser, Charles Ikenna Nwankwo, Ludger Herrmann, Joachim Müller

**Affiliations:** a Institute of Agricultural Engineering, Tropics and Subtropics Group, University of Hohenheim, Garbenstrasse 9, Stuttgart 70599, Germany; b Institute of Soil Science and Land Evaluation, University of Hohenheim, Emil-Wolff-Strasse 12a, Stuttgart 70599, Germany

**Keywords:** Agglomerator, Encapsulation, Encrustment, Pelletizer, Seed coating, Seed treatment

## Abstract

Seed granulation is a coating technique, which turns a raw material mixture of sand, loam, water, seeds, and fertilizers into seedballs. It enhances the seedling establishment and early growth of crops, like pearl millet, in nutrient-poor soil. Mechanization is highly required, as large-scale production poses challenges to local farmers due to time constraints and labor demand. The prototype of a drum granulator for seeds, also known as a seedball machine, essentially consists of a metal frame and a drum. The seedballs are formed by a rotational motion of the drum. The construction and operation of the machine were designed to be simple. In this study, the combined effect of different factors, such as substrate composition, rotational speed and residence time was taken into account. This study revealed that the amount of loam and the rotational speed of the drum appeared to be the most influencing factors on seedball production and quality. The machine had a production capacity of seedballs ten times higher than manual production. The machine-made seedballs were also of high quality, exceeding 98% germination rate under greenhouse conditions. Besides pearl millet, the machine can be potentially used for other small-sized seeds, such as cotton or sesame.

## Graphical abstract

**Figure:**
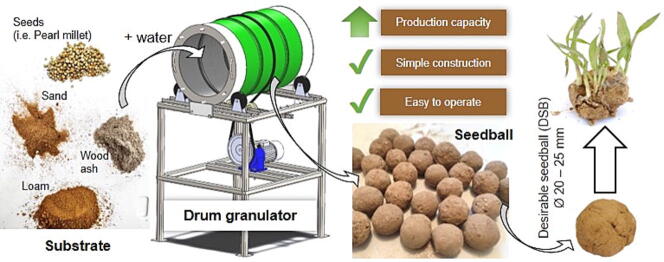

**Specifications table**

**Table:** 

Hardware name	Drum granulator for seeds – DG-SBM 2020
Subject area	• Engineering and Material Science • Agricultural Sciences
Hardware type	• Biological sample handling • Granulation of soil/seed-mixtures • Mechanization of seedball production
Cost of Hardware	Approx. 1400 €
Open Source License	CC BY 4.0
Source File Repository	https://doi.org/10.17632/t8bvm2cspk.1

## Hardware in context

Seed granulation or seedball making is a seed-pelleting technique, using locally available natural materials to improve crop establishment and early biomass production. Sand, loam, water, and seeds are the main raw materials. Nutrient additives are mandatory to achieve a positive effect. Other additives like pesticides are optional [1]. The materials are mixed into a substrate to which water is added and then molded into seedballs with a minimum diameter of 20 mm. The technique has been well-adapted to current seed sowing systems, particularly in sandy nutrient-poor areas with a tropical semi-arid climate, like the Sahel [2]. Ultimately, seedballs can evolve into a widely used practice in subsistence farming and rangeland management.

In the semi-arid Sahel region of Africa, the soil is often very nutrient-poor with low water holding capacity. Consequently, average crop yields are low [3]. Research has shown that sowing seedballs enhances plant development, in particular during the seedling establishment phase [2], [4]. In the Sahel, the major staple crop is pearl millet (*Pennisetum glaucum* [L.] R. Br.). Pearl millet is drought tolerant and responds well to the application of fertilizers [5]. Therefore, this crop was selected as the study object. Nevertheless, the technique can potentially be adapted to other crops with small-sized seeds like *Sorghum bicolor* or *Digitaria exilis* [1].

The major issue in manual seedball production is the labor-demand to mix and mold the raw material. Therefore, a machine prototype built to mechanize production should ensure proper and fast mixing, which can substantially increase production capacity while sustaining seedball quality in terms of germination rate.

The main principles for the machine construction are low cost and ease in production and operation. Focusing on these principles ensures the technical and economic feasibility of the machine’s application. Sticking to these principles also increases the chances that this innovation is adopted in smallholder agriculture, as well as it ensures successful technology dissemination to the target users [6]. For the time being, the main testers of this technology are farmers organized in cooperatives in the Maradi region of Niger. This paper presents the proof-of-concept of a prototype drum granulator for seeds to produce seedballs that was designed and constructed at the University of Hohenheim.

## Hardware description

A low-cost motor-powered drum granulator was designed, constructed and tested, in order to mechanize seedball production for smallholder farmers in tropical regions with nutrient-poor soils. The machine was designed under a sensible framework of low cost, reproducible and scalable construction, as well as ease of operation.

**Fig. 1:**
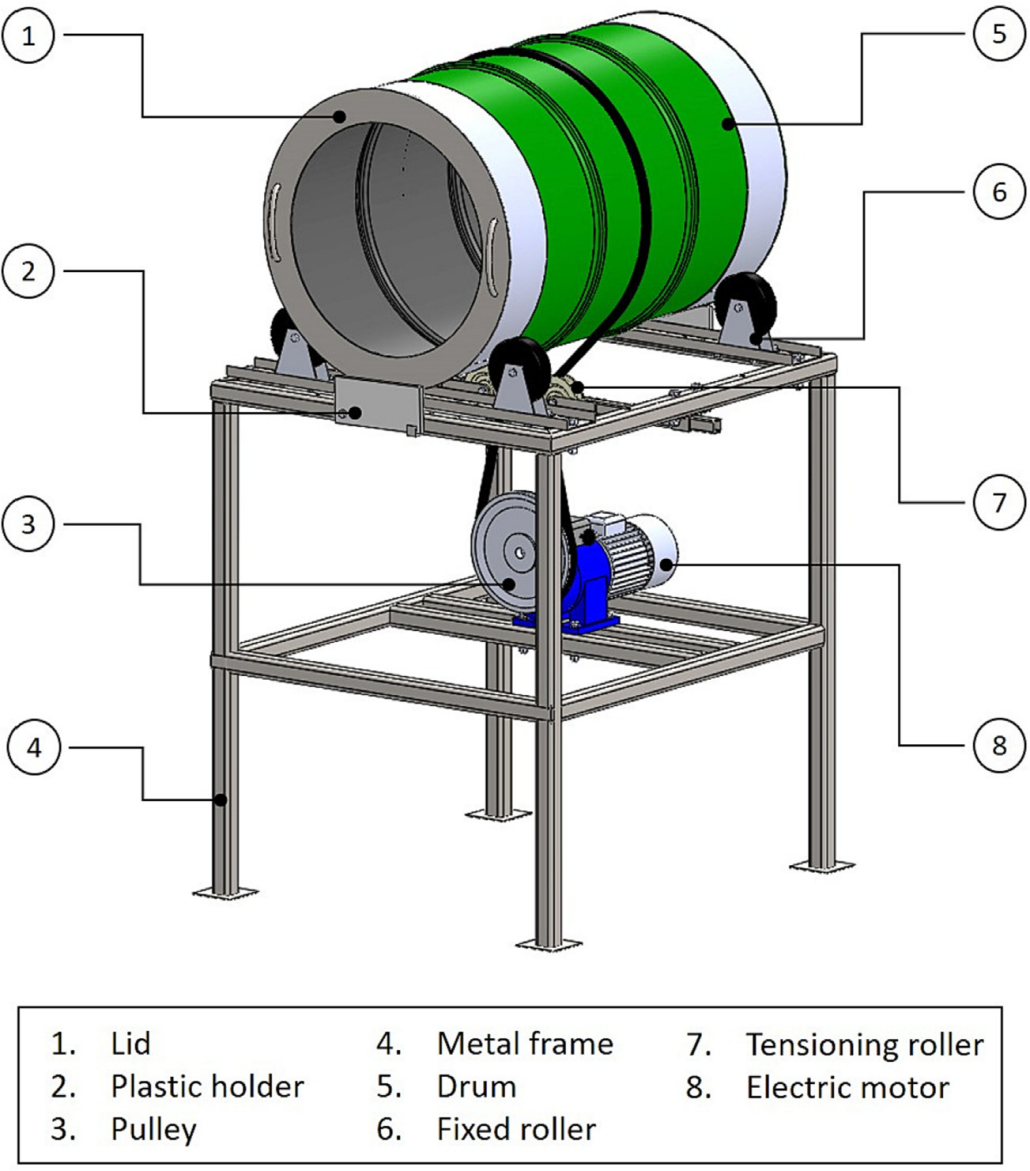
Three-dimensional model and the main parts of the prototype drum granulator for seeds (seedball machine).

The three-dimensional model of the machine is presented in Fig. 1. It consists of three main parts: (I) the drum for seedball formation, (II) the electric motor for rotating the drum and (III) the metal frame that holds the drum and the motor. The overall height, length and width of the machine are 1600, 900 and 800 mm, respectively.

### Drum (I)

The drum was placed on top of the metal frame on four fixed rollers (Bockrolle 125/37, SSI Schäfer Shop GmbH, Betzdorf, Germany). A 200-liter standard oil barrel, 600 × 900 mm was used as drum in the original machine because it is readily available worldwide. However, other sizes are also possible. A hollow removable lid was placed on the barrel to prevent material from falling out during production. Through the lid, operators must be able to access the material in the machine during production. Seedballs were formed, as all the raw materials were agglomerated in the drum through a rotational motion.

### The motor (II)

The electric motor (NRM-005/1, Tramec Getriebe GmbH, Lahr, Germany) was the most sensitive and most expensive part of the machine. It has 294 N torque and is equipped with a variable gear to adjust the drum’s rotational speed. Any electric motor can be basically used, as long as the motor has the capacity to rotate the drum at a maximum rotational speed of 50 rpm. The rotational speed of an electric motor can be adjusted either by diameter of the pulley, a reduction gearbox or a frequency converter connected to the motor.

### The metal frame (III)

The metal frame functioned as the main structure of the machine, where the drum, the motor and other mechanical and electronic systems, were attached. The frame was constructed of square metal tubes (25x25 mm; 30x30 mm; 40x40 mm), which were welded together for increased stability and robustness. It has a rectangular surface of 800 × 900 mm. The motor should be placed in the center of the metal frame, below the drum.

### Drive system and components (IV)

The system generates a rotational motion of the drum that is crucial to form seedballs from the substrate. The main components include fixed roller, holder rail, V-belt, pulley, tensioning roller and tachometer. The V-belt wraps around the drum and is connected to a drive pulley, which attached to the electric motor. A standard V-belt was chosen to allow the use of a standard drive pulley and standard tensioning rollers. The drum is placed on top of four fixed rollers attached on two holder rails at the front and back sides of the drum. As the motor runs, power is transmitted to the drum through the V-belt to create the rotational motion.

A common V-belt (B/17, optibelt GmbH, Carol Stream, Illinois, USA) and a V-belt pulley with a nominal diameter of 224 mm (Aluminum profile XPB, SPB and B (17), Mädler GmbH, Stuttgart, Germany) were used to connect the drum and the electric motor. On top of the metal frame, a digital tachometer (CF5135C-Z, Auto Leaders Co., ltd, Shenzhen, China) was attached to regularly monitor the rotational speed of the drum. Placed on top of the metal frame were also the holder rails, onto which the fixed rollers were screwed. The screw holes were placed 42 mm apart. The tensioning rollers were also added to adjust the tension of the V-belt. The tensioning roller consists of two parts; a telescopic holder, and a fixed tube, which was welded to the bottom of the upper metal square tubes on the left and right side of the metal frame. The roller was attached to a telescopic holder on the left and right side of the metal frame, which could be inserted into the fixed tube. The telescopic holder and the fixed tube were joined with a butterfly nut.

**Fig. 2:**
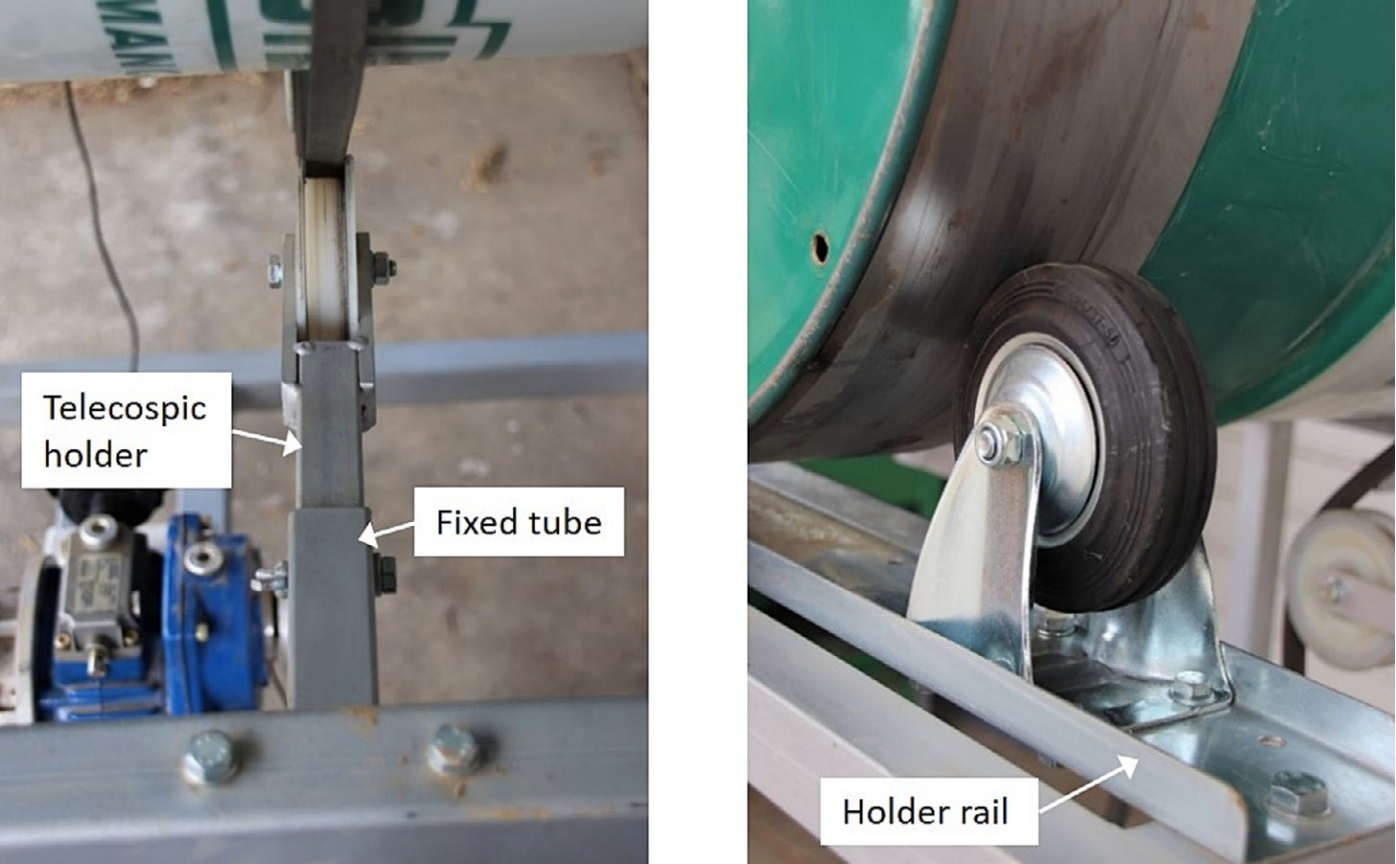
Special features of the drum granulator; a tensioning roller (left) and a fixed roller on a holder rail (right).

By placing multiple screw holes 20 mm apart on the telescopic holder, the length of the V-belt could be regulated. This allowed the users to choose from a wide range of V-belts lengths and types. On the other hand, the screw holes on the holder rails could adjust the distance of the fixed rollers, whereas drums with different sizes could be used. See Fig. 2. Since rotational speed of the drum was the important machine setting, a rotational speed sensor for the drum offered flexibility to use various types of electric motors and pulleys.

A sprayer is used for water application. It ensures a uniform water distribution, which is important to control the amount of water within the seedballs, and consequently the seedball quality.

**Fig. 3:**
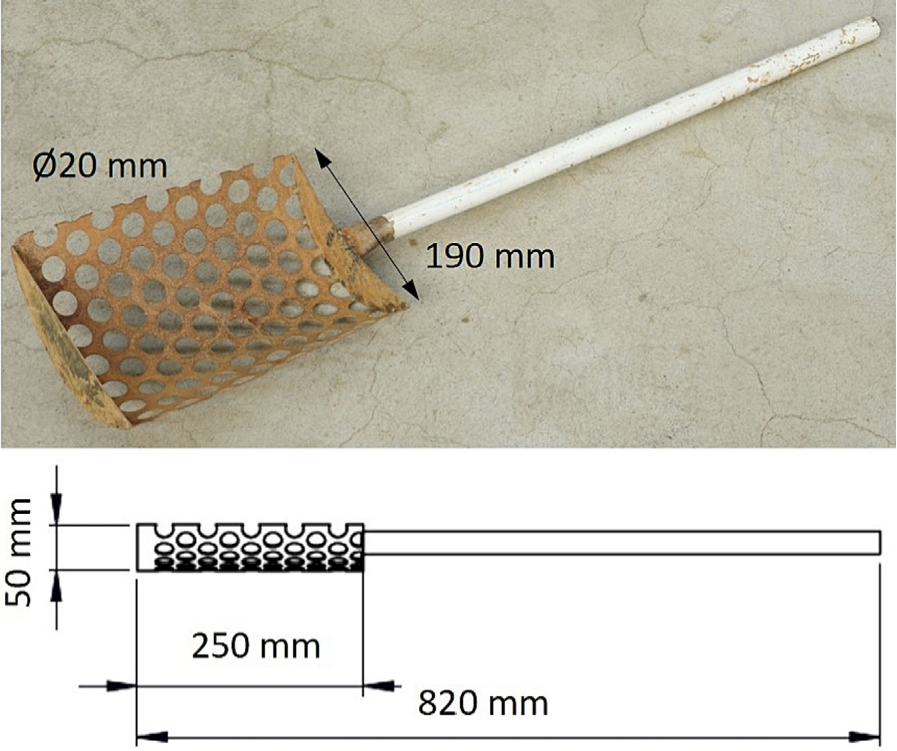
The perforated shovel.

Another vital tool was a perforated shovel, as presented in Fig. 3. It was designed to control seedball diameter and to separate and collect desirable seedballs (DSB). The shovel’s blade was bent into the inner shape of the drum. The front side of the blade shall be closed, in order to avoid any seedballs from falling out during separation. The hole diameter of the blade shall fit the minimum acceptable diameter of DSB, which is 20 mm. The overall length of the shovel was 820 mm.

## Design files

### Design files summary

**Table:** 

**Design file name**	**File type**	**Open source license**	**Location of the file**
SBM_2021_assembled.IGS	CAD file	CC BY 4.0	Source File Repository
SBM_2021_assembled.SLDASM	CAD file // Solidworks	CC BY 4.0	Source File Repository
SBM_partsZip_pdf	zip // adobe pdf part files	CC BY 4.0	Source File Repository
SBM_partsZip_SW	zip // solidworks part files	CC BY 4.0.	Source File Repository
Seedball Video	Mp4	CC BY 4.0	https://bit.ly/3v0aGkL

## Bill of materials

**Table:** 

**Designator**	**Component**	**Number**	**Cost per unit [€]**	**Total cost [€]**	**Source of materials**
Mainframe	Square tube (40x40) 12400 mm	1	3.18	93.44	https://bit.ly/3f9QXJF
	Square tube (30x30) 300 mm	1	4.21	4.21	https://bit.ly/3h34BAH
	Square tube (25x25) 6,000 mm	1	4.45	26.74	https://bit.ly/3eWqWxe
	Plastic sheet (Thickness = 10 mm)	2	3.54	7.08	https://bit.ly/3zHPvau
	Metal sheet 2000 × 1000 mm (1.5 mm thickness)	2	130.54	261.08	https://bit.ly/3GTvp0R
	Screw (M10) – 12 mm	9	0.23	2.07	https://bit.ly/3ek7Vpp
	Screw (M8) – 12 mm	32	0.1	3.20	https://bit.ly/3xO9kft
	Nut (M10)	8	0.04	0.32	https://bit.ly/2PPaw10
	Nut (M8)	37	0.03	1.11	https://bit.ly/3eY66gM
	Washer (M10)	17	0.03	0.51	https://bit.ly/2Rs2StO
	Washer (M8)	79	0.01	0.79	https://bit.ly/3tmQnNz
	Butterfly nut (M10)	1	0.18	0.18	https://bit.ly/3ugUBrg
	Butterfly nut (M8)	6	0.23	1.38	https://bit.ly/3b3n4t5
Mechanical system	Electric motor variable gear	1	767.24	767.24	Tramec Getriebe GmbH http://www.tramec-getriebe.de/
	Standard 200-L oil barrel	1	39.90	39.90	https://bit.ly/3aYkSms any standard 200-L oil barrel is suitable
	Belt pulley (SPB)	1	67.74	67.74	https://bit.ly/3BThC6A
	V-belt	1	26.7	26.70	https://bit.ly/3nTZOCR
	Fixed roller	4	12.79	51.16	https://bit.ly/3eh80tT
	Tensioning roller	2	11.25	22.50	https://bit.ly/3v2Pc6N
Electronic system	Digital tachometer	1	13.09	13.09	https://amzn.to/33g3W6T
Other	Water sprayer	1	92.02	92.02	https://amzn.to/2QTcGNx
**Estimated total cost**				1390.44 €	

### Building instructions

*With regard to machine stability and safety of the drum during operation, the following step-by-step instructions have been created:*

**Fig. 4:**
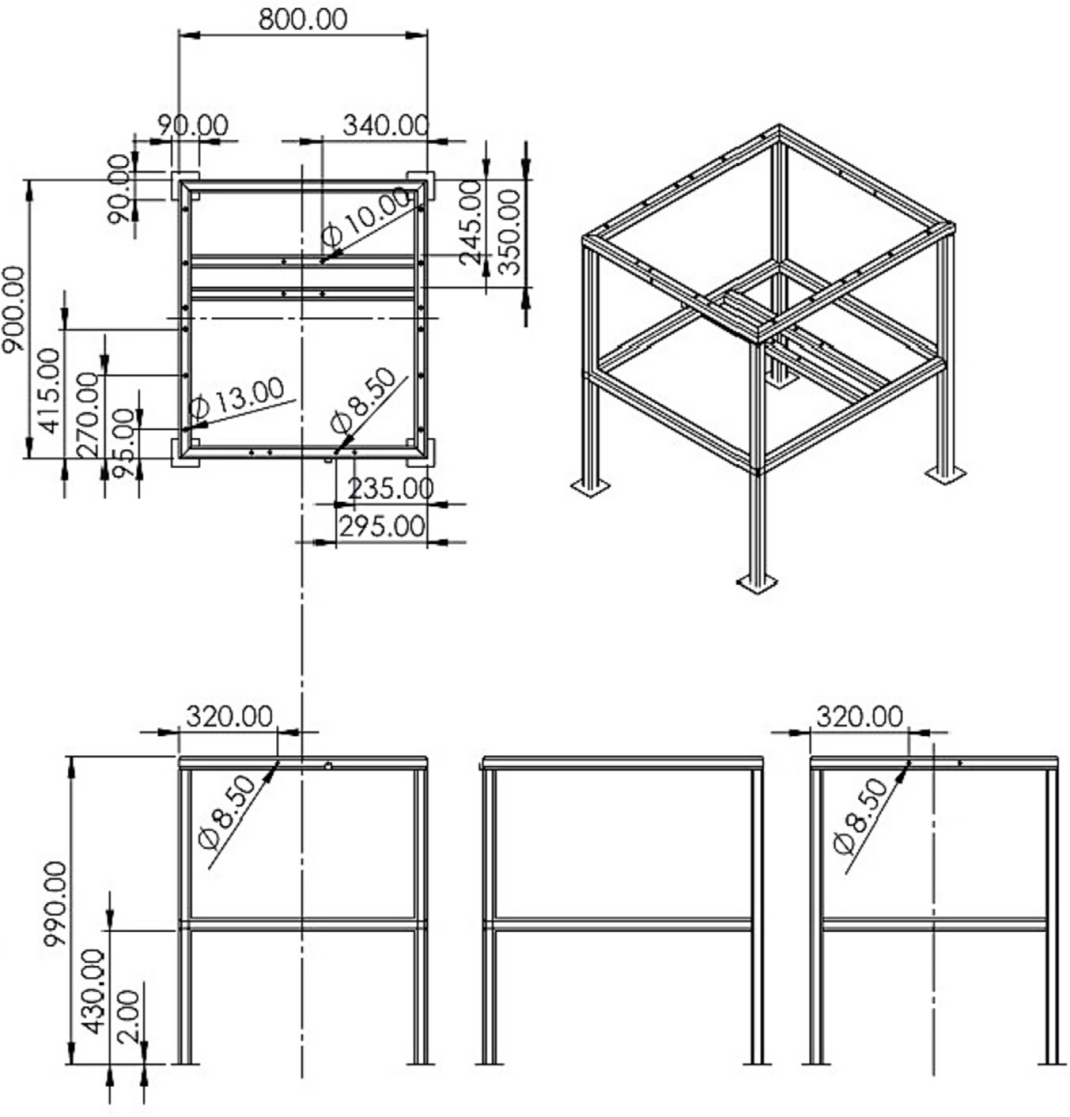
Metal frame of the prototype drum granulator (seedball machine). All dimensions are in mm.
C. Use a metal sheet, with a width of 130 mm to prepare the holder rails. Drill holes in the sheet, 42 mm apart, to connect the fixed rollers later. Afterwards, bend both sides of the sheet 90 degrees to create a rail. The walls of the holder rail should be about 20 mm high. Finally, attach the holder rails at each corner on the top of the mainframe. Screw the fixed rollers to the holder rails. In total, two holder rails are required at the front and back side of the drum.
D. The tensioning roller consists of two parts, a telescopic holder and a fixed tube, in which the holder is placed. The fixed tube is attached to the underside of the upper square tubes, placing the rollers in the middle of the tube. The telescopic holder is a smaller tube that can be placed inside the holder to adjust the length. Therefore, multiple holes need to be drilled in the square tubes, so the two parts can be joined. Attached to the smaller tube are two shorter extensions that hold the roller, which guides the V-belt. Two tensioning rollers are important to adjust different sizes of V-belts, which can be used for the machine.
E. Prepare a removable lid for the drum: The drum's lid is basically a disc-shaped steel plate with a minimum thickness of 2 mm, which has the same diameter as the outer diameter of the drum. On the backside of the lid, a round flange should be added to securely attach the lid to the drum. Therefore, the outer diameter of the flange must match the inner diameter of the drum, and the flange should reach at least 30 mm into the drum. Furthermore, two handlebars should be attached to the front of the lid, so the lid can be removed easily. A 120 mm wide strip of metal cover sheet must be wrapped around the drum, on the area where the drum lies on the fixed rollers. This is crucial to level any dents in the drum and to ensure smooth rotation.
F. Select the electric motor and pulley. The electric motor must have a torque of at least 20 Nm, with a maximum of 200 rpm of the motor. Place the motor in the middle in such a way that the belt pulley is in the center of the metal frame below the drum. Fix it to the metal frame.
G. Position the drum on top of the structure. Make sure the wheels of the fixed rollers fit the size of the drum.
H. Put the V-belt around the pulley and drum for power transmission. Adjust the tension of the V-belt by tightening the tensioning rollers.
I. After the drum is in place, screw two plastic holders into the metal frame. One in front of the machine and one in the back. This holder will keep the drum in place during operation.
J. Set the rotational speed of the drum with a digital tachometer.
K. Construct the perforated shovel by drilling 20 mm holes into a metal sheet with a minimum thickness of 1 mm. Then bend the metal sheet to approximately the same curvature as the interior of the drum. Close the front and back of the shovel by welding a piece of metal to it. Then attach a socket and shaft to the shovel.

A. Firstly, the barrel for the drum needs to be chosen, as its size determines many of the machine’s dimensions. Prepare all other materials, according to the bill of materials.
B. To build the metal frame, first cut the metal square tubes into usable pieces. Drill holes into the square tubes, where necessary. These drill holes are required to attach the other parts later. Then construct the metal frame by welding together the 40 × 40 square tubes, as outlined in Fig. 4**.** Start with the outer parts, then add the middle level. At the base of the machine, metal stands should be added to stabilize and level the machine. All metal parts should be finished before preparing the mechanical and electrical systems. After the construction of the metal frame, it should be protected with an anti-rust coating.

## Operation instruction

The operating procedure outlined in this chapter is based on previous research [2], [7] and targets seedballs with a diameter between 20 and 25 mm referred to as desirable seedballs (DSB).

### Substrate preparation

Sand and loamy soil are the major raw materials required for seedball production, as they contribute to more than 90% of substrate composition. To ensure good machine performance and seedball quality, sand and loam must be sieved to remove any coarse particles. Ideally, pearl millet seeds are the largest single particles in the mixture of raw materials. If wood ash is added, it should also undergo the same pre-treatments. For further application, any additives like NPK fertilizers can also be added without any additional treatments.

Based on the previous research [2], [7] and several preliminary experiments in this study, the recommended substrate composition for germination of pearl millet seeds is 60% sand, 35% loam, (2% of wood ash or 1% NPK mineral fertilizer) and 4 % seeds. This mixture can be further modified depending on local conditions in other regions. However, it is important to note that any additional osmotic compounds (e.g. wood ash, NPK, in particular ammonia compounds) are reducing the germination rate. Therefore, when changing the compounds, germination tests are mandatory before large-scale application. For an efficient production, it is recommended to prepare all the substrates in separate containers before feeding them into the machine.

### Operation procedure

Seedballs gradually accumulate substrates, as they are rolled. Therefore, the drum granulator is semi-continuously operated. The basic principle is that only seedballs, which have passed the size criteria of 20 mm diameter, were separated and collected with the perforated shovel. Afterwards, a new batch of substrate and water was added to continue the granulation process with the remaining materials from the previous round.

**Fig. 5:**
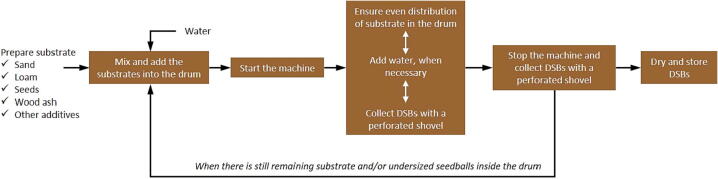
Operation instruction of the drum granulator (seedball machine).

The procedure is also outlined in Fig. 5. A proper amount of substrate shall be added every time. A production cycle was divided into consecutive rounds (normally between 6 and 10 rounds), where each round included adding substrate and water, running and stopping the machine, as well as collection of DSB. Afterwards, it is recommended to dry the collected DSB at a temperature of 40 °C for 48 h.

To start an operation, a substrate (including the seeds, without water), which had been previously prepared, should be spread along inside the drum to avoid any bulking. The ideal starting load for the 200-L barrel used in this study is 1000 g of substrate. A water sprayer is used to apply water to the substrate in the drum and to control the amount. Based on our preliminary observation, it is imperative not to add too much water at once, as this will have adverse effects on seedball formation. The material should be moist, but not soaked.

Once the machine is switched on, the substrate is moved and mixed in the drum. Simultaneously, water is occasionally added using a water sprayer. After a while, the materials start to accumulate and form undersized seedballs. If there is no longer any substrate visible in the machine and the first seedballs have visually reached about a diameter of 20 mm, the machine should be stopped. All the DSB are collected with a perforated shovel, and new substrate is added. About 80% of the starting load is recommended, and the substrate shall be distributed evenly. The first and second rounds will normally take longer than the subsequent ones. Afterwards, the machine is turned on again and the next round begins. More water can be added, if necessary. Once seedballs are formed, production is stopped to collect the DSB and to add a new substrate. Usually, this will be the case after 1 to 2 min. These steps are repeated for each round until there are no undersized seedballs left in the drum. At this point, the process is started over again. During the first rounds, only a few DSB are generated. Nevertheless, more and more DSB are ready to be produced in the subsequent rounds.

**Fig. 6:**
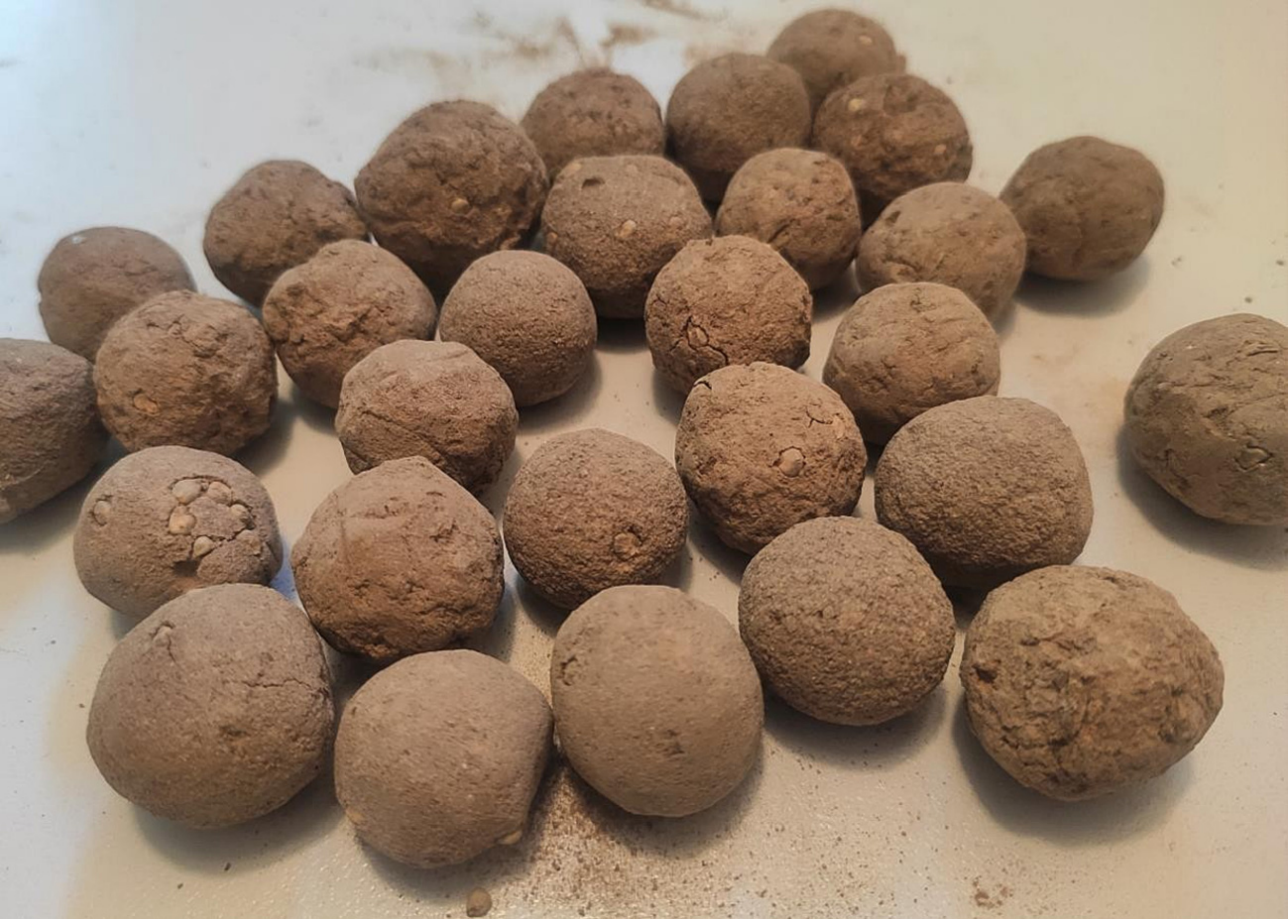
Dried desirable seedballs (DSB) collected with the perforated shovel.

Ultimately, it is up to the operator of the machine to observe the process and to decide when to separate and collect the DSB, to add new substrate and/or water, as well as to stop the machine. This might require some training and time to develop the know-how for operating the machine. After production, seedballs should be directly dried to prevent any unwanted germination. Fig. 6 presents dried DSB, which were ready for sowing.

### Safety measures

Overall, the operation of the machine is safe. However, some precautions must be taken. Operators should avoid touching any moving parts during production, especially the V-belt. It is also important to make sure clothing or hair does not get tangled up in the drum when removing seedballs while the drum rotates. If any fertilizers, pesticides or other additives are used, the respective safety requirements must also be considered.

## Validation and characterization

### Effect of operational settings on seedball production

The machine production capacity was characterized by the number of DSB (diameter between 20 and 25 mm) produced per minute (DSB/min). Substrate usage rate (SUR) described the percentage of substrate turned into DSB. Machine efficiency was calculated by the dry mass of the total DSB produced divided by the total mass of substrate. Three main factors, such as the amount of loam in substrate composition, rotational speed and residence time, could affect the production capacity, substrate use and quality of the seedballs. These parameters were optimized in a scientific experiment using response surface methodology with Box-Behnken design. The seedballs were classified into different sizes using sieves with different hole diameters.

**Fig. 7:**
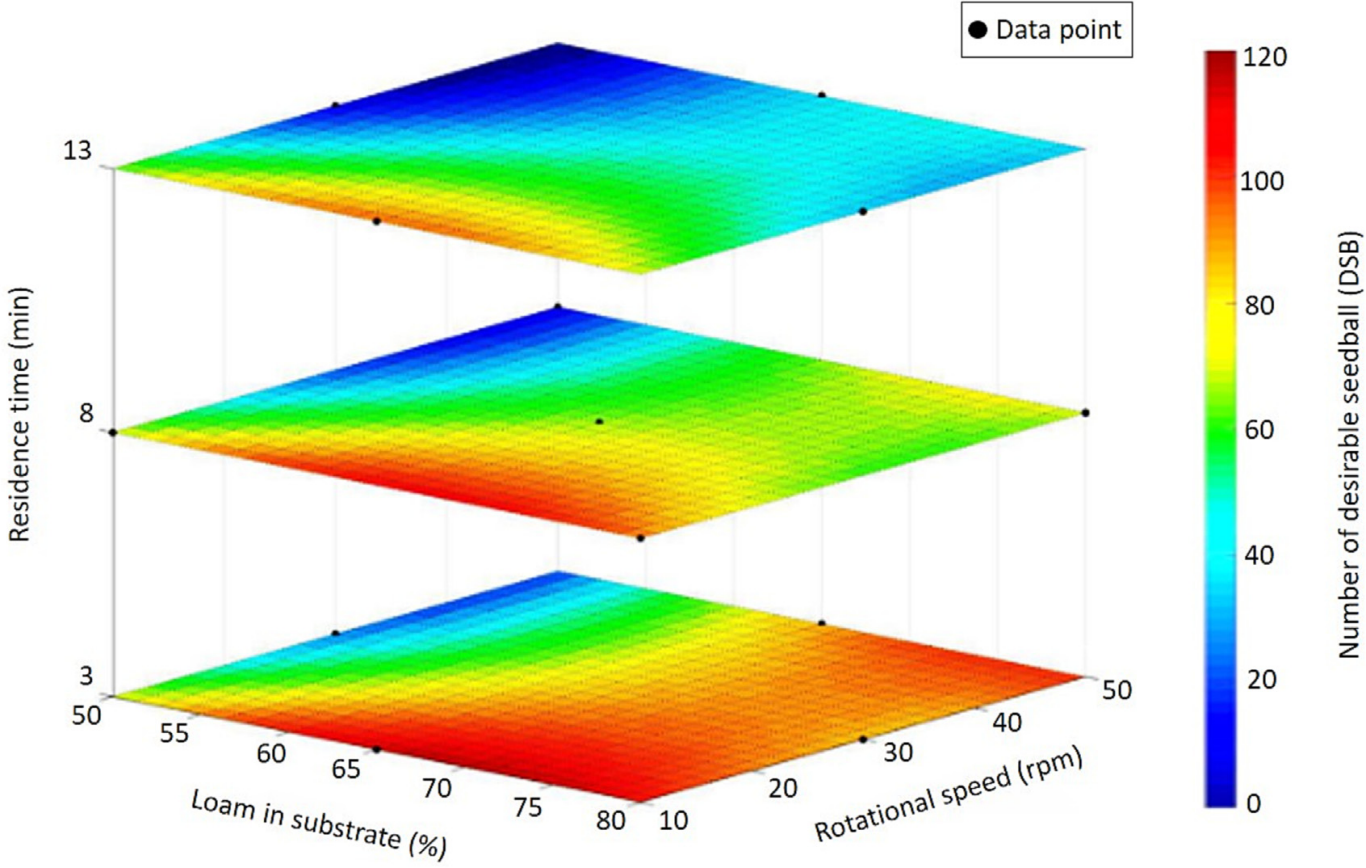
A sliced plot of the operational setting effects (amount of loam in a substrate, rotational speed, and residence time) on the number of desirable seedballs (DSB).

Fig. 7 visualizes the effect of the three variables in a sliced response surface plot. The results from this scientific experiment were specific to the raw materials used in this study. Statistical analysis of the model using a least significant difference (LSD) test shows that the amount of loam in the substrate, the rotational speed of the drum and the residence time of the material in the drum had a significant effect on the number of DSB at *p* ≤ 0.05. Moreover, the interactions among the factors, for instance, residence time and rotational speed, had a significant influence on the DSB production at *p* ≤ 0.05. Overall, the model was in good agreement with an *R^2^* of 0.872. Lack-of-fit was insignificant at the 95% confidence level, and the MAPE value was 12.68% [8].

Table 1 presents the ANOVA table of the corresponding model. Among all the factors, the loam seemed to be the most influential one. A likely explanation is that loam acted as a binding agent. Therefore, seedballs accumulated new material faster with high loam contents in the mixture. Rotational speed and residence time also had significant impacts at *p* ≤ 0.05. The constant rotational speed of the machine ensured that the raw materials were properly mixed. Faster rotational speed and longer residence time would lead to lower number of DSB. At high rotational speeds, the substrate agglomeration process tended to be faster. Therefore, the DSB should be taken out at the right time, before they become too large. Due to the self-reinforcing relationship between the two parameters, the rotational speed of the drum should be fixed, while the residence time of the substrate could be adjusted for each round. In further research, rotational speeds lower than 10 rpm should be included and the underlying reasons of physical relationships explaining the effects should be investigated.

**Table 1:** ANOVA for the quadratic model concerning operational setting effects on the number of desirable seedballs (DSB).

**Source**	**Sum of squares**	**DoF**	**Mean square**	***F*-value**	***p-*value**
Intercept	10913.22	5	2182.64	10.40	0.0007*
Loam in substrate	2628.13	1	2628.13	12.53	0.0046*
Rotational speed	2520.50	1	2520.50	12.01	0.0053*
Residence time	2145.13	1	2145.13	10.22	0.0085*
Lack-of-fit	1240.71	7	177.24	0.6643	0.7018
Pure error	1067.30	4	266.82	–	–
Correction total	13221.24	16	–	–	–

* significant at *p* ≤ 0.05.

### Production capacity – Application tests

Application tests were conducted to evaluate the machine’s performance in practice. For the machine to be viable, production capacity should greatly exceed those of manual production, while the seedball quality should be at least similar. The maximum loading capacity of the machine was 2000 g. Table 2 shows a comparison of all relevant parameters in manual and mechanized production. The table presents the output advantage of mechanized production. Compared to manual production, a sufficient number of seedballs could be produced to sow one hectare in 3.2 h instead of 40 h. This represented a substantial time saving of more than 90%. Using the operating procedure outlined in the previous chapter, the output was 44 ± 6 DSB/min. The average production cycle was eight rounds with an average time of 1.7 min and 76 DSB/round.

**Table 2:** Comparison of manual and mechanized seedball production (Average value).

**Parameter**	**Mechanized production**	**Manual production**
Production of desirable seedballs (DSB/min)	44	4
Number of seeds/DSB	28	22
Coefficient of variation seeds/DSB (%)	15.5	7.5
Substrate usage rate (%)	94.8	95.0

**Fig. 8:**
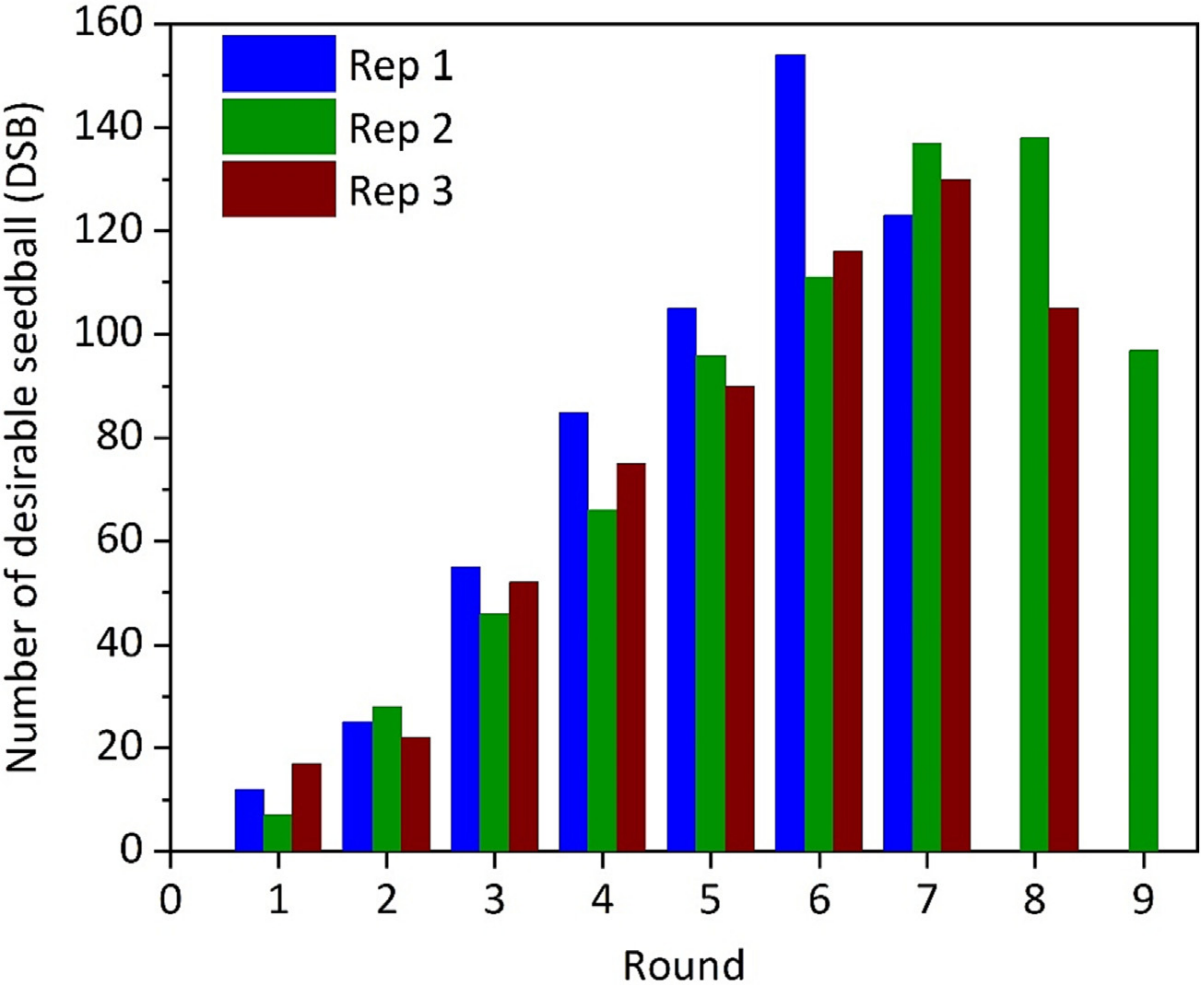
The number of desirable seedballs (DSB) produced per round under the optimized operational setting with repetitions (Rep).

Fig. 8 shows how the number of DSB collected in a round changed from the first to the last round of a production cycle. In the early rounds, only a few DSB were produced. On the other hand, undersized seedballs were also formed. During these early rounds of production, the total number of seedballs and the amount of substrate (new and remaining one) in the drum increased. From the second round onwards, an increase of DSB production could be observed, as more seedballs started to fulfil the diameter criteria.

In the following rounds, the amount of substrate loaded in the drum started to decline, as more DSB were collected than new substrate added. Consequently, the number of DSB began to level off, before declining. Other important parameters, such as the number of seeds and the coefficient of variation of seeds per seedball, were not influenced by the production rounds. There was no indication that semi-continuous production would affect the utilization of seedballs.

The validation tests showed that the machine could also be used efficiently under some modifications in the substrate composition. This is an important feature, as the composition might need to be changed to cater to the specific demands of other seeds or regions. Table 3 presents several performance parameters from manual and mechanized seedball production in different substrate compositions. During the validation runs, the average substrate usage rate was 94.8 ± 2.9 %. Substrate usage rate is a crucial parameter, and it should be maximized, as the sourcing of raw materials is the most expensive aspect of seedball production [2].

**Table 3:** Performance of the drum granulator under different substrate compositions.

**Parameter**	**Trial run 1**	**Trial run 2**
	**(74% loam, 26% sand)**	**(35% loam, 65% sand)**
Number of rounds	9	8
Total run time (min)	14	13.5
Number of DSB	726	572
Substrate usage rate (%)	97.9	90.3
Production (DSB/min)	51.8	42.4
Production (DSB/Run)	80.7	71.5
Unit mass (g/DSB)	9.9	10.4

As previously presented in Fig. 7, higher loam contents in the substrate led to better machine performance. However, in any scenario, the machine fulfilled the basic benchmarks of substrate usage rate and production capacity. Therefore, the percentage of loam in the substrate could be adjusted to agronomic needs, rather than the production requirements. In terms of machine setting, the loam share was related to the rotational speed of the drum. On the other hand, the amount of loam also had an influence on the germination rate of seeds in a seedball.

### Power consumption

Power consumption is another important variable that influences the machine implementation in remote and potentially off-grid rural regions. Power consumption was measured, using a modular energy measurement tool, adapted to low voltage networks (Janitza UMG 96 RM, Janitza electronics GmbH, Lahnau, Germany). With 1,300 g machine load and 50 rpm, the average power consumption was modest, ranging from 140 to 145 W. Statistical analysis also shows a significant correlation between speed and power consumption at *p* ≤ 0.05. With increased loading capacities, there would be an increase in power requirements.

**Fig. 9:**
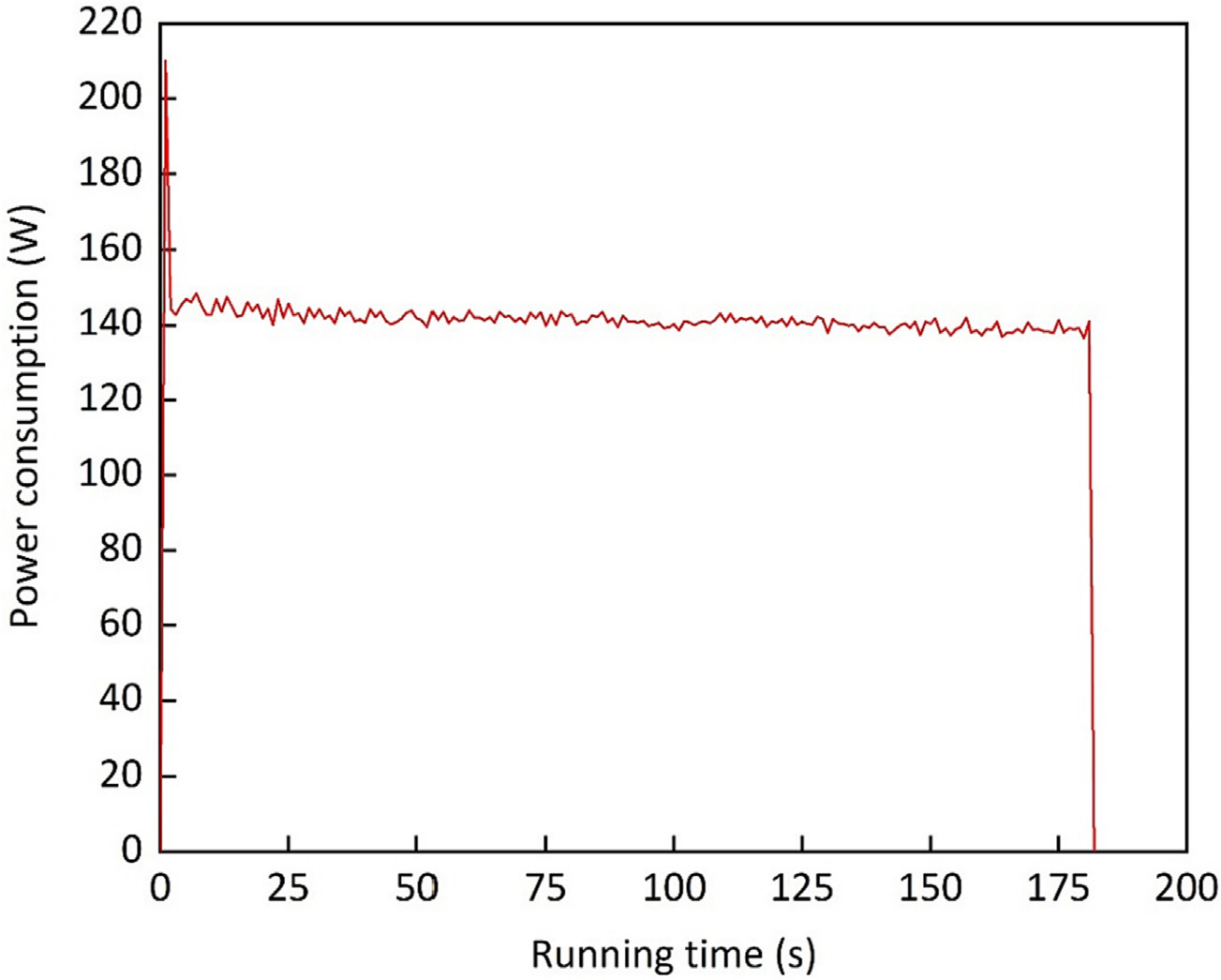
Power requirement of the drum granulator over time during seedball production.

Fig. 9 presents the development of power requirements over time. There was a peak at 210 W, as production begins. Immediately afterwards, the average power consumption dropped to 141.2 W. Moreover, there was a slightly negative trendline with advancing runtimes. It appears that less power was necessary to rotate the machine, once seedballs were formed.

This moderate power consumption shows that it was possible to operate the machine with a variety of power sources, like solar radiation, wind or a generator set. The findings also show that it would be possible to power the machine with a photovoltaic (PV) system, which would allow the machine to operate independently in off-grid regions.

### Seedball quality

The mechanized seedball production should have a similar seedball quality as the manual production, in order to be viable. The seedball quality parameters included the number and coefficient of variation of seeds per seedball, rupture force, unit mass and geometrical characteristics. The geometrical characteristics of DSB were measured using a Vernier digital caliper (Series 500, Mitutoyo Deutschland GmbH, Neuss, Germany). The validation runs averaged 28 ± 8 seeds/DSB, which was slightly higher than what was found in manual production (22 seeds/DSB). Nevertheless, the number of seeds in a seedball could be adjusted by increasing or decreasing the number of seeds in the substrate. With a proper machine setting, coefficient of variation of the number of seeds per seedball was reduced from 17.59% to 7.9%. The coefficient of variation might also be reduced, because the seeds were also added gradually, just like the other raw materials. Rupture force was measured using a laboratory texture analyzer (Instron 3400 Series, Instron Inc., Norwood, MA, US). During characterization, tests rupture force ranged between 85.1 N and 186.9 N. Rupture force should not be too high, as it might have adverse effects on germination. The average diameter and unit mass of a DSB was 22.5 ± 2.5 mm and 9.5 ± 0.7 g, respectively. Statistical analysis indicated that the machine's operational settings significantly influence the rupture force at *p* ≤ 0.05.

Other geometrical characteristics, such as sphericity and unit volume, were also calculated according to Karaj and Müller [9]. The average sphericity and unit volume of a DSB were 0.86 ± 0.05 and 31.16 ± 4.81 mm^3^, respectively. However, the findings show that those physical parameters were not suitable to describe seedball quality. Therefore, the diameter was the only relevant physical size criteria.

### Germination test of seedballs

**Fig. 10:**
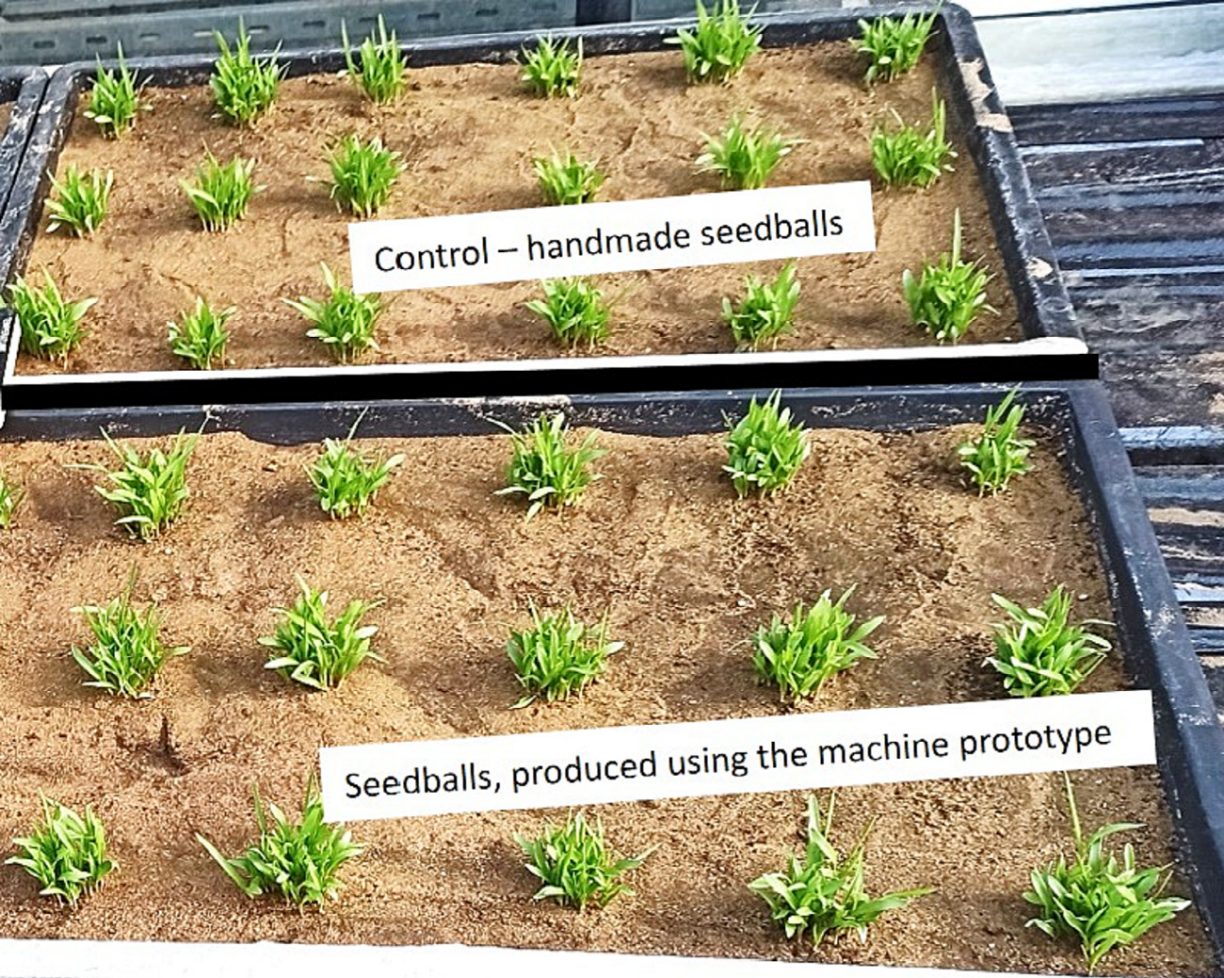
A germination experiment of seedballs made by hand and with the drum granulator under greenhouse conditions at the University of Hohenheim, Germany.

The germination rate of the seeds in the seedballs produced from the machine is also an important criteria [10]. Ideally, 90% (or more) of the seeds in a seedball would germinate. Germination tests of seedballs from manual and mechanized production were conducted in a research greenhouse, as shown in Fig. 10. On average, 17 ± 6 pearl millet seeds out of about 25 seeds germinated per seedball during the germination test. This was estimated by breaking up the seedballs and manually counting the seeds. In general, only one of the 72 seedballs used in the experiment did not germinate. The germination rate was 98.6%, which was similar to the seedballs from manual production. Based on the data, it appears that the seeds were not damaged in a way that would reduce their ability to germinate.

### Outlook

The drum granulator, also called a seedball machine, has proven to be a successful concept for a viable mechanized seedball production. Further on-site tests are necessary to test the machine performance under real operating conditions. Simultaneously, suitable business models, which support the dissemination of the machine, need to be developed and implemented in cooperation with the stakeholders. The potential for further applications of the machine shall be explored, as follows:

• Seedball production with other seeds, like sesame, sunflower or amaranth
• Enhancement of substrate composition with biochar, pesticide, commercial fertilizers and soil improvers.

## Declaration of Competing Interest

The authors declare that they have no known competing financial interests or personal relationships that could have appeared to influence the work reported in this paper.
